# Understanding the psychological impact of the climate crisis on individuals with depression: a phenomenological study

**DOI:** 10.1038/s41598-026-39907-4

**Published:** 2026-02-11

**Authors:** Cemile Hurrem Ayhan, Özge Sukut, Sakine Aktaş, Mehmet Cihad Aktaş, Seda Karakaya Cataldas, Uğur Ozkan

**Affiliations:** 1 Faculty of Health Science, Department of Psychiatric Nursing, Van Yuzuncu Yil University, Van, Turkey; 2 Department of Psychiatric and Mental Health Nursing, Istanbul University-Cerrahpasa Florence Nightingale Nursing Faculty, Istanbul, Turkey; 3 Faculty of Medicine, Department of Pscyhiatry, Van Yuzuncu Yil University, Van, Turkey; 4 Faculty of Nursing, Department of Psychiatric Nursing, Koc University, Van, Turkey; 5 Instıtute of Health Science, Department of Psychiatric and Mental Health Nursing, Van Yuzuncu Yil University, Van, Turkey; 6 Faculty of Health Science, Department of Psychiatric Nursing, Yuzuncu Yil University, Van, Turkey; 7 Faculty of Health Science, Van Yuzuncu Yil University, Campus, Tusba, Van, 65080 Turkey

**Keywords:** Climate change, Climate crisis, Mental disorders, Depression, Health care, Psychology, Psychology

## Abstract

Climate change-related weather events have profound effects on human health, including mental well-being. Research indicates that the climate crisis contributes to various psychological disorders, such as depression and anxiety. Understanding how individuals diagnosed with depression experience and interpret the impact of climate change can help in developing preventive, protective, and therapeutic interventions for this vulnerable group. This study explores the experiences of individuals with depression regarding the climate crisis, their perceptions of its effects on their illness, and their needs for possible solutions. Using a phenomenological approach, in-depth interviews were conducted with twelve participants. The data were collected through a personal information form and a semi-structured interview, focusing on participants’ views and experiences related to the climate crisis. Thematic analysis revealed five key themes: (1) Effects on Daily Life and Social Functioning, (2) Health Impacts, (3) Climate Anxiety and Future Concerns, (4) Emotional and Psychological Responses, and (5) Coping Mechanisms and Recommendations. Findings suggest that individuals with depression experience multidimensional negative effects due to the climate crisis, affecting their well-being and daily lives. There is a pressing need to develop targeted interventions that enhance psychological resilience in this group, helping them cope with the ongoing and future challenges posed by climate change.

## Introduction

Climate change, often referred to as the climate crisis, is defined as long-term alterations in global temperature patterns, precipitation, and the frequency of extreme weather events, largely driven by anthropogenic greenhouse gas emissions^1^. Over the past century, global average surface temperatures have increased by approximately 1.1 °C above pre-industrial levels, with recent decades marked by accelerated warming, more frequent heatwaves, floods, droughts, and ecosystem degradation^1^. These environmental changes represent one of the most pressing global challenges of the 21 st century, affecting ecological systems, economies, public health, and social stability worldwide^2^.

Beyond its physical and environmental consequences, the climate crisis has profound implications for human life and well-being. Climate-related disruptions influence livelihoods, food and water security, housing stability, and social cohesion, thereby increasing exposure to chronic stressors and uncertainty^3^. Such widespread disruptions contribute to psychological strain even among individuals who are not directly exposed to extreme weather events, underscoring the pervasive nature of climate-related stress in contemporary societies^4^.

An expanding body of research demonstrates that climate change is closely linked to adverse mental health outcomes. Acute events such as floods, wildfires, and heatwaves, as well as subacute and chronic environmental changes, have been associated with increased rates of depression, anxiety, post-traumatic stress, sleep disturbances, and psychological distress^5,6^. Population-level studies further indicate that periods of extreme heat are correlated with higher rates of emergency department visits for mental health crises, suggesting a direct relationship between climate-related stressors and worsening psychiatric outcomes^7^. In parallel, the concept of climate anxiety has gained increasing attention, describing persistent worries, feelings of helplessness, and emotional distress related to climate change, which have been observed across diverse age groups and cultural contexts^8,9^.

Individuals with pre-existing mental health conditions appear to be particularly vulnerable to the psychological impacts of climate change. Growing empirical evidence indicates that people living with mental disorders often experience more severe symptoms, reduced coping capacity, and heightened vulnerability during climate-related disruptions^10^.

Within this broader group, individuals diagnosed with Major Depressive Disorder (MDD) stand out as especially at risk. Depression is commonly associated with difficulties in emotion regulation, diminished psychological resilience, and negatively biased cognitive appraisals—features that may amplify sensitivity to climate-related stressors (American Psychiatric Association, 2022). Recent studies suggest that climate-related stress does not simply coexist with depression but may be experienced as aggravating depressive symptoms, deepening feelings of hopelessness, and further undermining psychological well-being among individuals with MDD^11,12^.

Despite growing quantitative evidence linking climate change and mental health, there remains a notable lack of qualitative research exploring how individuals with MDD subjectively experience the climate crisis in their everyday lives. Understanding how people with depression perceive climate-related threats, interpret environmental changes, and integrate these experiences into their emotional and cognitive worlds is essential for developing person-centered interventions. Moreover, healthcare professionals increasingly recognize the importance of addressing climate-related concerns within mental health care, emphasizing the need to integrate environmental stressors into clinical assessment and treatment planning^13,14^.

In response to these gaps, this study aims to explore the lived experiences of individuals diagnosed with Major Depressive Disorder (MDD) and how they perceive the impact of the climate crisis on their mental health and everyday lives in Van, Turkey. Van is located in Eastern Turkey and is marked by a harsh continental climate, a strong reliance on agriculture and animal husbandry, and relatively limited socioeconomic resources compared to the western regions of the country^15^. These contextual conditions were considered important for understanding how people living with depression experience, interpret, and respond to climate-related challenges in their daily lives. To better understand these experiences, the present study focuses on individuals diagnosed with Major Depressive Disorder, allowing for a more focused exploration of climate-related experiences within a clearly defined clinical group.

Adopting a qualitative approach, the study seeks to gain an in-depth understanding of how climate-related stressors are perceived, emotionally experienced, and managed by individuals with depression. By focusing on lived experiences rather than symptom severity or causal attributions, the findings aim to contribute to the growing literature on climate change and mental health, while also offering insights that may inform clinical practice and policy initiatives aimed at strengthening psychological resilience among vulnerable populations in the context of ongoing climate change.

## Method

Throughout this study, the Consolidated Criteria for Reporting Qualitative Research (COREQ) were followed^16^.

### Study design

This study employed a phenomenological approach within the qualitative research paradigm to explore the lived experiences of individuals diagnosed with MDD in the context of the climate crisis. A phenomenological design was chosen because it allows for an in-depth understanding of participants’ subjective experiences and perceptions. Data were analyzed using reflexive thematic analysis as described by Braun and Clarke (2006), which facilitated the identification of themes and patterns emerging from participant narratives. This method was selected due to its flexibility in capturing the depth of lived experiences in qualitative research^17,18^.

### Participants

Participants consisted of 12 individuals diagnosed with MDD, recruited using purposive sampling. Recruitment took place between February and May 2024 at the psychiatric outpatient clinics of Van Yuzuncu Yil University Dursun Odabaş Medical Center. Major Depressive Disorder is known to be associated with heightened sensitivity to seasonal changes, environmental conditions, and disruptions in circadian rhythms, all of which are closely related to climate-related stressors. For this reason, the study focused on a clinically more homogeneous group of individuals diagnosed with MDD, allowing for a clearer exploration of how climate-related experiences were perceived and interpreted within this specific clinical context.

Given the phenomenological focus of the study, the aim was not to reflect the full epidemiological complexity of depression, but to gain a coherent understanding of lived experiences. Although psychiatric comorbidity—particularly anxiety disorders—is common in MDD, participants with additional psychiatric diagnoses were excluded to reduce analytic complexity and to better distinguish experiences primarily related to depression, especially in relation to climate anxiety. This decision was made to support conceptual clarity rather than to minimize the clinical relevance of comorbidity.

The inclusion criteria were: (a) being 18 years of age or older; and.

(b) having received an MDD diagnosis at least one year prior, ensuring that participants had experienced a prolonged course of the disorder and were able to reflect on the longer-term impact of the climate crisis on their mental health.

### Diagnosis verification and clinical characteristics

All participants had previously been diagnosed with Major Depressive Disorder by a psychiatrist at Van Yuzuncu Yil University Dursun Odabaş Medical Center as part of routine clinical care. No additional structured diagnostic interviews or symptom severity scales were administered by the research team, as the focus of the study was on participants’ lived experiences rather than on symptom comparison or measurement.

At the time of the interviews, participants were receiving outpatient psychiatric follow-up. Information on current symptom severity or medication use was not systematically collected, as variations in treatment and symptom levels were considered part of participants’ everyday experiences of living with depression rather than factors to be controlled.

The exclusion criterion was the presence of any psychiatric comorbidity (e.g., bipolar disorder, schizophrenia, or anxiety disorders), as the study aimed to focus specifically on the experiences of individuals with MDD without potential confounding influences from additional psychiatric conditions.

Data collection was guided by the principle of data saturation. Consistent with phenomenological methodology, data saturation achieves between the 9 and 17 interviews^19^. Saturation was reached at the tenth interview, when recurring patterns and themes began to emerge and no new information was identified. This was further confirmed during the eleventh interview, and one additional interview was conducted to ensure analytical rigor. Data collection was therefore concluded after the twelfth interview.

A total of 12 individuals were approached and informed about the study. All individuals who were approached met the eligibility criteria, provided informed consent, and completed the interviews. No participants withdrew from the study.

The participants (six males and six females) aged 21–55 years (M = 34 years). Demographic characteristics, including marital status, education level, and duration of depression, are summarized in Table 1.

**Table 1:** Characteristics of participants.

Demographic characteristics	
Age	
Range	21 to 55 years
Average participants age	34 years
Male average age	35 years (DP1, DP3, DP4, DP7, DP8, DP9, DP10, DP11, DP12)
Female average age	31 years (DP2, DP5. DP6)
Marital status	
Married	6 participants (DP2, DP3, DP4, DP8, DP10, DP11)
Single	6 participants (DP1, DP5, DP6, DP7, DP9, DP12)
Education status	
Bachelor	6 participants (DP1, DP4, DP5, DP8, DP9, DP12)
High school degree	6 Participants (DP2, DP3, DP6, DP7, DP10, DP11)
The length of depression (Years)	
1–3 Years	10 Participants (DP1, DP2, DP3, DP4, DP5, DP6, DP7, DP9, DP11, DP12)
4–6 Years	2 Participants (DP8, DP10)

### Data collection tools

The study data were collected using a personal information form and a researcher-developed semi-structured interview guide through individual in-depth interviews. The personal information form gathered data on participants’ sociodemographic characteristics and illness-related information related to Major Depressive Disorder.

A researcher-developed semi-structured interview guide was used in this study. The guide consisted of open-ended questions designed to explore participants’ views, perceptions, and lived experiences regarding the climate crisis and its impact on their mental health and daily lives. To ensure alignment with the study objectives and content relevance, the first author conducted preliminary consultations with psychiatric nursing scholars, clinicians specializing in mental health and mood disorders, and individuals diagnosed with depression.

Prior to the main data collection, the interview guide was pilot tested with two individuals who met the inclusion criteria but were not included in the final sample. Based on feedback obtained from these pilot interviews, minor revisions were made to improve the clarity, depth, and comprehensibility of the questions.

The main interview questions are presented in Table 2.

**Table 2:** Interview questions.

No.	Interview question
1	How do you perceive the climate crisis, and what does it mean to you personally?
2	In what ways, if any, do you think the climate crisis affects you in your daily life?
3	How do you think the climate crisis influences your mental health or emotional well-being?
4	Can you describe situations in which climate-related issues (e.g., extreme heat or environmental changes) have affected your mood, thoughts, or behaviors?
5	How do you think the climate crisis influences the symptoms and course of your illness over time?
6	What emotions do you experience when thinking about climate change and the future?
7	How do you cope with situations or concerns related to the climate crisis that affect you negatively?
8	Is there anything else you would like to share about how the climate crisis affects your mental health or life experiences?

### Study procedure and ethical considerations

#### Participant recruitment

Potential participants were identified through referrals from mental health professionals working at the psychiatric outpatient clinics of Van Yuzuncu Yil University Dursun Odabaş Medical Center. These professionals provided patients with information about the study, and individuals who expressed interest were subsequently contacted by the research team for eligibility screening. Written and verbal informed consent was obtained from all participants prior to their inclusion in the study.

#### Interview process

Interviews were conducted face-to-face in a private room within the psychiatric outpatient clinic to ensure confidentiality and participant comfort. Each interview lasted between 18 and 42 min (M = 30 min). While the semi-structured interview guide provided a general framework, interviews were conducted flexibly. Probing and follow-up questions were used throughout the interviews to clarify participants’ responses, encourage elaboration, and deepen understanding of their experiences (e.g., “Can you tell me more about that?” or “How did that affect you at the time?”). Additional questions were asked when necessary to explore emerging issues and ensure that participants’ meanings were fully captured.

All interviews were conducted in Turkish, the participants’ native language. The interview transcripts were translated into English by the first author, who is fluent in both Turkish and English and has experience in qualitative research and academic writing. To ensure translation accuracy and conceptual equivalence, translated excerpts were reviewed by a second bilingual researcher. Discrepancies were discussed and resolved through consensus to preserve the original meaning of participants’ narratives.

With participants’ permission, all interviews were audio-recorded, and field notes were taken to capture non-verbal cues and contextual observations. Audio recordings were transcribed verbatim and cross-checked against the original recordings for accuracy. Participants were offered the opportunity to review their transcripts for validation and to provide corrections or additional comments.

### Ethical considerations

Ethical approval was obtained from the Van Yuzuncu Yil University Non-Invasive Clinical Research Ethics Committee (Protocol No: 2023/11–24, Date: 10/11/2023). Institutional permission was also granted by Van Yuzuncu Yil University Dursun Odabaş Medical Center (Protocol No: 478324, Date: 23.01.2024). The study adhered to the ethical principles outlined in the World Medical Association Declaration of Helsinki.

Given the sensitive nature of the interview topics and the vulnerability of the study population, specific measures were taken to minimize potential distress during data collection. Interviews were conducted by researchers with clinical experience in mental health, who were attentive to participants’ emotional states throughout the interview process. Participants were informed that they could pause, skip questions, or terminate the interview at any time without providing a reason.

If signs of emotional distress emerged during an interview, the interviewer responded supportively, offered breaks, and assessed whether the participant wished to continue. Following the interviews, participants were reminded of their ongoing access to routine psychiatric care through the outpatient clinic. When appropriate, participants were encouraged to discuss any distress arising from the interview with their treating mental health professionals. No interviews were terminated due to distress, and no adverse events were reported during the study.

### Reflexivity and research team

The research team was made up of six members with clinical and academic experience in mental health: faculty members from psychiatric nursing and one faculty member with a medical background in psychiatry. All researchers had previously worked with individuals diagnosed with depression and other mental health conditions. This shared clinical background inevitably shaped how the team approached the study, making them particularly attuned to stories of distress, vulnerability, and challenges related to symptoms and treatment.

From the beginning, the team was aware that their professional training could lead them to interpret participants’ accounts through a clinical lens, potentially emphasizing impairment or psychopathological aspects of experience. There was also an initial assumption that climate-related stressors might worsen depressive symptoms or interfere with recovery. Rather than trying to set these assumptions aside completely, the researchers engaged with them reflexively, paying close attention to how such perspectives might influence the way interviews were conducted, how data were coded, and how themes were developed.

Reflexive journaling was used throughout the research process as a way of recording researchers’ thoughts, emotional reactions, and emerging interpretations. These reflections were regularly revisited during team discussions to help ensure that the analysis remained closely grounded in participants’ own accounts, rather than being shaped primarily by clinical expectations or diagnostic frameworks.

Field notes were written during and immediately after the interviews to capture non-verbal cues, emotional tone, pauses, and contextual details. Although these notes were not coded as data in their own right, they were used to enrich and clarify the interpretation of the interview transcripts—particularly in moments where meaning was communicated through affect, silence, or interaction rather than words alone.

Reflexivity was further supported through ongoing discussions within the research team, where differing interpretations were openly shared and negotiated. In addition, an external psychiatrist with experience in qualitative research independently reviewed the coding framework and thematic structure. The level of agreement between the research team and the external reviewer was 85%, providing an additional layer of critical reflection and strengthening the credibility of the analysis.

### Data coding and theme development

The data analysis process followed Braun and Clarke’s (2006) reflexive thematic analysis approach, allowing for an in-depth examination of participants’ narratives. Although the study was grounded in a phenomenological qualitative perspective, reflexive thematic analysis was chosen as the analytic approach because it offered both flexibility and depth in exploring how participants experienced and made sense of the climate crisis. This method enabled us to remain attentive to individual lived experiences while also identifying shared patterns across accounts.

The phenomenological orientation of the study informed both data collection and interpretation by placing participants’ subjective meanings and first-person perspectives at the center of the analysis. Reflexive thematic analysis was used to organize and interpret these lived experiences, rather than to impose rigid or abstract categories that might distance the analysis from participants’ own words. Themes were therefore developed to reflect the core meanings of participants’ experiences in a way that remained faithful to their narratives^17^.

**Fig. 1:**
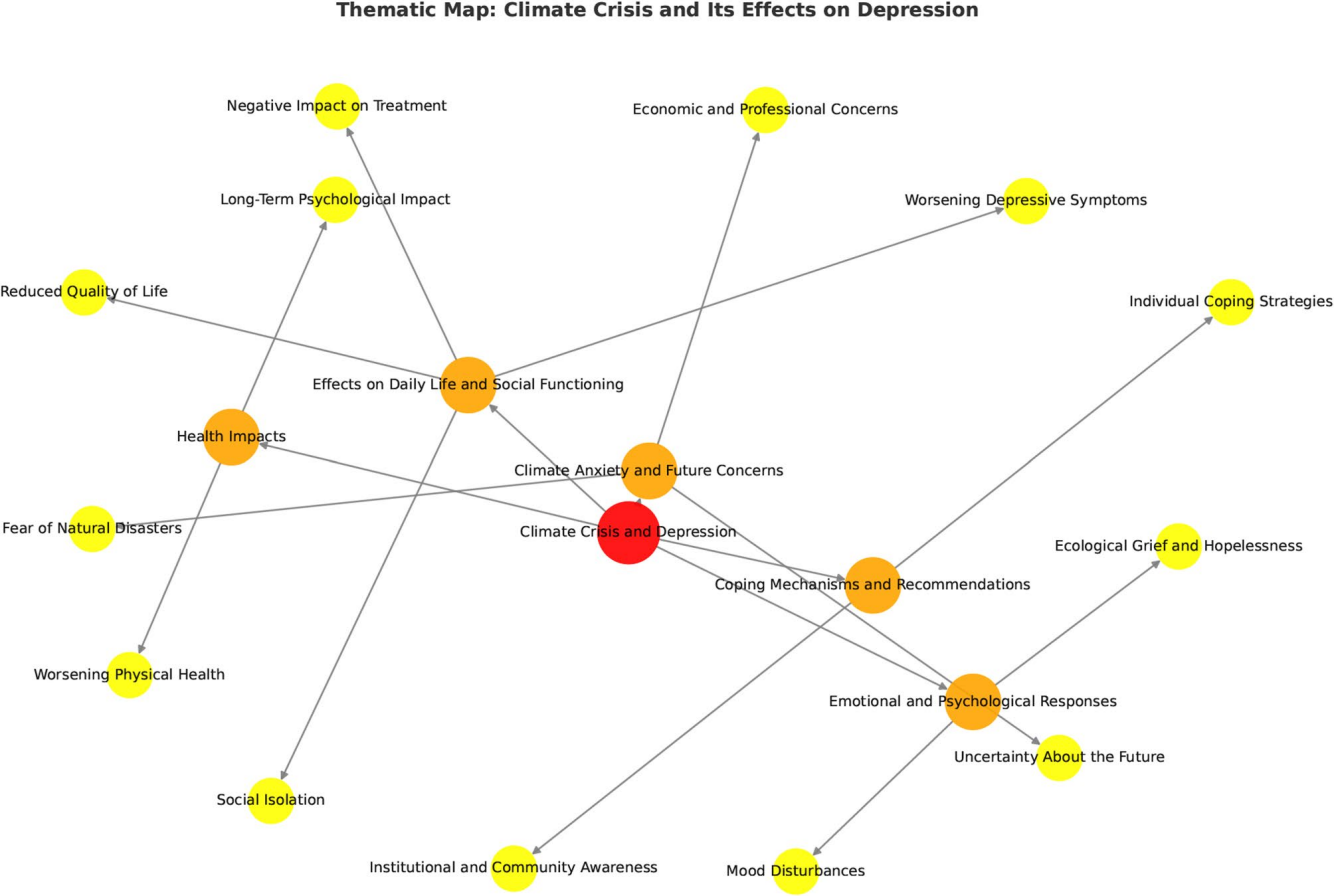
Thematic map.

Analysis was conducted in a series of systematic and iterative stages:

1. Familiarization with the Data: All interviews were transcribed verbatim and reviewed multiple times to ensure accuracy. Researchers engaged in repeated reading of the transcripts to become immersed in the data and to identify preliminary patterns of meaning.
2. Generating Initial Codes: Using MAXQDA qualitative data analysis software, the transcripts were systematically coded. Initial coding was carried out independently by two researchers with training in qualitative methods. An open coding approach was used, allowing meanings and concepts to emerge directly from the data rather than being guided by predefined categories. Coding focused on participants’ explicit expressions and experiences, attending primarily to semantic content rather than deeper latent interpretations.
3. Searching for Themes: Codes were examined and grouped into broader conceptual categories, leading to the identification of candidate themes and subthemes. This stage involved collaborative discussions among the research team to ensure that emerging themes were conceptually coherent and aligned with the study objectives.
4. Reviewing and Refining Themes: Preliminary themes were reviewed in relation to both the coded extracts and the dataset as a whole to ensure that they meaningfully reflected participants’ lived experiences. Points of overlap, ambiguity, or difference in coding and theme boundaries were discussed reflexively among the researchers until shared understanding was reached. Through this iterative process, themes were refined to improve clarity, internal coherence, and distinction, in line with the principles of reflexive thematic analysis.
5. Defining and Naming Themes: Final themes and subthemes were clearly defined and named to reflect their core meanings. Detailed analytic descriptions were developed to capture the essence of each theme and illustrate how they related to the overarching phenomenon under investigation.
6. Development of the Thematic Map (Fig. 1**)**: Based on the finalized themes and subthemes, a thematic map was developed to visually represent the relationships among the identified themes. Figure 1 illustrates this thematic structure using a network-style layout, in which themes are displayed as nodes and conceptual connections between themes are represented by linking lines. This visualization is intended as an interpretive aid to enhance understanding of thematic interrelationships, rather than as a standalone analytic technique or a formal network analysis.

The color scheme in Fig. 1 reflects levels of thematic abstraction. The central focus of the study (*Climate Crisis and Depression*) is shown in red, higher-order themes are presented in orange, and subthemes are displayed in yellow. The size and color of nodes do not indicate statistical weight, frequency, or network centrality. Instead, the figure serves as a conceptual representation of qualitative findings.

The thematic map was created using visualization software to organize themes derived from qualitative analysis. No formal network analysis metrics (e.g., centrality measures or weighted edges) were applied. This approach aligns with qualitative methodological literature that prioritizes conceptual clarity and relational interpretation over quantitative modeling^20^.

7. Ensuring Trustworthiness: To enhance credibility, member checking was conducted by inviting participants to review preliminary interpretations of the findings. In addition, an external qualitative researcher independently reviewed the coding framework and thematic structure. Agreement was calculated as percentage agreement based on consistency in code–theme alignment and theme identification, resulting in an agreement rate of 85%. No inter-rater reliability coefficients (e.g., Cohen’s kappa) were calculated, as reflexive thematic analysis emphasizes reflexive consensus and interpretive coherence rather than statistical reliability.

To ensure research trustworthiness, the study followed the criteria established by Lincoln and Guba^21^:

- Credibility: Transcripts were cross-checked with recordings, returned to participants for verification, and themes were compared across interviews.
- Transferability: Detailed methodological descriptions were provided to allow replication in different contexts.
- Dependability: Reflexive journaling was employed to maintain self-awareness and minimize researcher bias.
- Confirmability: The study followed an inductive approach, deriving themes directly from the data rather than preconceived theoretical frameworks^21^.

This study employed a phenomenological approach as its theoretical framework and reflexive thematic analysis as its methodological approach. The sampling criteria ensured that participants had sufficient lived experience of MDD (at least one year) while minimizing potential confounders by excluding individuals with psychiatric comorbidities. Ethical guidelines were strictly followed, and strategies were implemented to enhance credibility, confirmability, and transferability in qualitative research. These methodological refinements strengthen the study’s contribution to understanding how individuals with MDD experience and cope with the effects of the climate crisis.

## Results

This study explores the experiences of individuals diagnosed with MDD regarding the effects of the climate crisis. Through this systematic approach, five key themes emerged from the analysis: (1) Effects on Daily Life and Social Functioning, (2) Health Impacts, (3) Climate Anxiety and Future Concerns, (4) Emotional and Psychological Responses, and (5) Coping Mechanisms and Recommendations (see in Fig. 1).

### Theme 1: effects on daily life and social functioning

Participants described how climate-related changes—particularly extreme heat and unstable weather—had gradually become woven into their everyday lives. Rather than being experienced as occasional external stressors, these conditions were described as shaping daily routines, social interactions, and emotional states, often in ways that intensified existing depressive symptoms.

*Social isolation* emerged as a central experience. Many participants explained that rising temperatures and physically uncomfortable conditions reduced their motivation to leave home, leading them to withdraw from social life. This withdrawal was often described in bodily and emotional terms, as if the environment itself made participation in everyday life feel overwhelming. One participant expressed this physical and emotional burden clearly:

*“I hate the heat. It overwhelms me. Even now*,* I’m sweating.” (DP7)*.

Another participant described a sense of confinement that accompanied this withdrawal:

*“I feel trapped. I don’t want to go outside; I don’t want to leave the house.” (DP5)*.

Several participants highlighted the importance of companionship, particularly during periods of emotional vulnerability, noting that being alone often intensified negative thoughts:

*“I try to move to cooler places to protect myself from the heat. When someone is with me*,* I feel better. When you’re alone*,* bad thoughts increase.”* (DP8).

For several participants, these experiences of withdrawal were not limited to physical discomfort but were closely linked to broader worries about *declining living conditions and reduced quality of life*. Concerns about scarcity, economic insecurity, and unequal access to basic resources often merged with depressive thought patterns, reinforcing feelings of hopelessness and catastrophic expectations about the future. As one participant explained:

*“It affects me psychologically in a bad way. That’s why I don’t want to leave the house; I usually prefer to stay inside. These weather changes really upset me… It feels like the end of the world. There will be famine. We won’t be able to access food easily anymore… Life is already hard*,* and only those with money will be able to get things. When I think about this*,* I feel even worse.” (DP4)*.

Participants also described how climate-related stressors contributed to the *worsening of depressive symptoms*, which then further disrupted daily functioning and social relationships. Extreme heat was frequently linked to increased irritability, emotional reactivity, and withdrawal, making interpersonal interactions more strained:

*“As the weather gets hotter*,* I feel angrier. I shout at my family; I don’t want to talk to anyone.” (DP9)*.

A particularly salient pathway through which climate conditions affected daily life was sleep disturbance, which participants perceived as having a direct negative impact on both mood and recovery. Difficulty sleeping because of heat left participants feeling exhausted and emotionally depleted, making it harder to maintain routines or engage in treatment effectively:

*“I can’t sleep at night because of the heat*,* and when I don’t sleep*,* my depression gets worse.” (DP2)*.

Another participant similarly described how seasonal changes disrupted sleep and triggered a relapse in symptoms:

*“Summers are very difficult for me. It gets extremely hot*,* more than before—it wasn’t like this in the past. I think it’s because of climate change. I can’t sleep at night*,* and when I don’t sleep*,* my illness starts again.” (DP11)*.

Daily mood fluctuations were also linked to weather conditions, with some participants noting increased withdrawal during gloomy or foggy days:

*“My mood can change during the day. On cloudy and foggy days*,* I become more withdrawn.” (DP3)*.

Concerns about *treatment effectiveness* and recovery also featured prominently. Some participants feared that the cumulative psychological burden of climate-related stressors might interfere with their ability to manage depression or benefit fully from treatment.

*“If this continues*,* we may not be able to receive proper treatment. I can’t cope with the effects it creates. It prevents my recovery.” (DP5)*.

Despite the predominance of negative experiences, participants’ accounts were not entirely uniform. A small number described moments of partial relief or adaptation that helped counterbalance isolation and functional decline. Some noted that specific activities or conditions allowed them to reconnect, even temporarily, with daily life:

*“I usually don’t feel like doing much when the weather is gloomy*,* but when I go on nature walks*,* I actually enjoy it.” (DP1)*.

Similarly, one participant described maintaining social contact despite a general preference for staying indoors:

*“These changes in nature and seasons really demoralize me. I try to cope with these problems. I usually like spending time at home*,* but I still meet my friends. We play games together*,* and they make me feel better.” (DP12)*.

Taken together, these accounts illustrate how participants experienced climate-related changes as closely intertwined with their depressive symptoms, shaping social isolation, symptom severity, quality of life, and engagement with treatment. While many described withdrawal and difficulties maintaining everyday functioning, others shared moments of connection, coping, and adaptation. This variation highlights that climate-related stressors were not experienced in a single, uniform way but were lived, interpreted, and managed differently across participants’ daily lives.

### Theme 2: health impacts

Participants described the climate crisis as affecting both their physical and mental health in ways they experienced as closely connected. These health-related impacts clustered around two interrelated areas: *the worsening of physical health* and *long-term psychological effects*. Participants often interpreted these changes through the lens of living with depression, which shaped how bodily discomfort, emotional distress, and treatment-related challenges were understood.

Participants with chronic physical conditions, particularly respiratory illnesses, described increased sensitivity to environmental changes associated with the climate crisis. Rising temperatures, air pollution, and unstable weather patterns were perceived as directly aggravating physical symptoms and increasing vulnerability to illness.

*“I get sick more easily because of weather conditions. I have asthma*,* and cold weather really affects me. Climate change increases the likelihood of getting sick*,* especially for people with weak immune systems.” (DP6)*.

Another participant linked environmental pollution to broader physical health risks:

*“Factory emissions and car exhaust damage the air and nature. Unfortunately*,* this also increases the risk of asthma.” (DP9)*.

For these participants, physical health deterioration was not experienced in isolation but as part of an ongoing struggle that interacted with their mental health and daily functioning.

In addition to physical health concerns, participants emphasized the longer-term psychological impact of the climate crisis. Many described climate-related stressors as intensifying depressive symptoms such as hopelessness, emotional instability, withdrawal, and loss of pleasure. Exposure to distressing climate-related information, especially through media, was often experienced as emotionally overwhelming and difficult to regulate.

*“There are so many things caused by the climate crisis. Sometimes I see them on the news and feel hopeless” (DP9)*.

Another participant highlighted the depth of emotional exhaustion and disengagement associated with these experiences:

*“I don’t feel well at all. I don’t want to do anything. Whatever I do feels pointless. I don’t enjoy anything anymore. It’s like I’m living on my own*,* detached.” (DP11)*.

Beyond present symptoms, participants expressed anticipatory concerns about the future, often projecting their own experiences onto society more broadly. These reflections were marked by a sense of inevitability and collective vulnerability.

*“I’m normally an unhappy person*,* but these environmental factors make it worse over time. I think climate change will increase my depression in the future. I believe many people will become unhappy*,* and patients like me will increase.” (DP4)*.

Taken together, these accounts illustrate how participants perceived the climate crisis as exerting both immediate and cumulative effects on health. Physical deterioration and long-term psychological strain were experienced as intertwined processes, embedded within broader concerns about treatment, recovery, and future well-being.

### Theme 3: climate anxiety and future concerns

Participants described climate-related anxiety as a persistent and emotionally demanding experience marked by fear, uncertainty, and negative expectations about the future. Rather than being limited to immediate environmental changes, these concerns were woven into how participants thought about their own lives, their families, and the continuity of life across generations. Climate change was often perceived as an uncontrollable and potentially irreversible process, which contributed to a deep sense of existential insecurity.

A central aspect of participants’ accounts involved profound *uncertainty about the future*. Many expressed fears that ongoing environmental deterioration would make it difficult—or impossible—to plan in a meaningful way. This uncertainty was not described as a distant or abstract concern, but as an ongoing psychological burden that reinforced depressive thoughts and feelings of hopelessness.

*“If this continues*,* there will be no future for us.” (DP5)*.

For some participants, uncertainty stemmed from visible disruptions in natural cycles. Changes in seasons, altered weather patterns, and environmental degradation were experienced as signs that the world had become unstable and unpredictable, undermining feelings of safety and control.

*“Summers are getting hotter*,* but winter no longer has the same snow… It feels like the world is losing its balance.” (DP1)*.

Economic and occupational insecurity further intensified future-related uncertainty, particularly among participants whose livelihoods depended directly on environmental conditions. Individuals working in agriculture, construction, or outdoor labor described climate change as a direct threat to their ability to sustain themselves and their families.

*“If summers get even hotter*,* what will happen to our crops? How will we survive? This crisis affects not just me*,* but the future of my children as well.” (DP4)*.

Similarly, another participant emphasized how seasonal instability disrupted work life and amplified worries about long-term security:

*“Climate change affects us construction workers too. Seasonal changes disrupt our work… I think about what kind of life I’ll be able to provide for my children in the future. Climate change makes this much harder.” (DP12)*.

Future concerns were frequently framed in intergenerational terms. Participants often spoke not only about their own uncertainty but also about the anticipated impact of climate change on their children and future generations, which added a moral and emotional weight to their anxieties.

*“It will definitely affect my future and my family’s future negatively. If climate change is not stopped*,* we won’t even be able to talk about a future.” (DP5)*.

Alongside uncertainty about the future, participants described a form of *ecological anxiety* grounded in lived, everyday observations of environmental change. Those with close connections to land and nature spoke in concrete terms about declining agricultural productivity, water scarcity, and ecosystem loss. These changes were interpreted as early signs of broader social disruption and scarcity.

*“We have a garden*,* but it doesn’t produce fruit like before. I associate this with climate change… lakes drying up*,* fewer trees*,* less oxygen. It’s like we live winter in summer and summer in winter now. We can’t get fruits and vegetables like before… it feels like scarcity is starting.” (DP1)*.

For some participants, ecological anxiety extended into catastrophic future scenarios involving natural disasters, resource conflicts, and societal breakdown. These fears were often expressed with a strong emotional focus on children and future generations rather than on personal survival alone.

*“As a farmer*,* I look at it this way: if summers are dry*,* what will we plant? How will we survive? Climate change doesn’t only affect us but our children and future generations. It’s not just seasons changing—there will be melting glaciers*,* storms*,* disasters*,* water crises*,* even wars. People may not be able to access clean water… I worry more about my children than myself.” (DP4)*.

Taken together, participants’ narratives portray climate anxiety as a multifaceted experience shaped by environmental, economic, familial, and existential concerns. These anxieties were not abstract or hypothetical; they were rooted in lived observations, personal responsibilities, and everyday realities. For participants living with depression, climate-related uncertainty and ecological anxiety deeply influenced how they imagined the future and assessed the sustainability of life conditions.

### Theme 4: emotional and psychological responses

Participants spoke about a wide range of emotional and psychological reactions to the climate crisis, often describing these feelings as deeply entangled with their experience of depression. Rather than experiencing climate-related distress as a separate or temporary emotional response, many described it as something that settled into and intensified an already vulnerable emotional state. Feelings such as restlessness, pessimism, sadness, emotional fatigue, and deep hopelessness were frequently mentioned.

For several participants, climate-related changes contributed to a sense of emotional numbness and a fading sense of future direction. They described how ongoing environmental deterioration reinforced feelings of emptiness, loss of meaning, and reduced expectations from life, making it harder to imagine a hopeful or purposeful future.

*“Whether it rains or the sun comes out doesn’t matter to me. I always feel the same. I don’t feel good at all… I don’t think I have a future. I have no expectations from life. The climate crisis affects not only me but the whole world.”* (DP11).

Others described a more affectively charged response, marked by restlessness, inner tension, and emotional discomfort. Climate change was experienced as a source of psychological unease rather than fear alone:

*“There is a kind of discomfort that comes from people’s indifference to what is happening. The part of the climate crisis that affects me is this constant restlessness.”* (DP5).

The emotional burden of environmental destruction and the gradual loss of natural resources left some participants feeling deeply saddened and emotionally affected. Participants experienced *ecological grief and hopelessness.* Participant’s statements as follows:

*“Seeing the forests burning and the extreme heat breaks my heart… It feels like there is nothing beautiful left in the world.” (DP6)*.

Similarly, another participant noted:

*“With climate change*,* trees burning and the weather becoming extremely hot affect us deeply. It makes us sad… I feel very overwhelmed*,* and I don’t expect good things in the future.”* (DP6).

Weather conditions were also described as emotionally significant, particularly in interaction with depressive vulnerability. Participants perceived that adverse weather intensified depressive affect and emotional heaviness:

*“When the weather is bad*,* people feel more overwhelmed. Depression already pushes you into despair*,* and the weather really plays an important role in a person’s life.”* (DP12).

Overall, participants’ accounts show that their emotional responses to the climate crisis went beyond feelings of anxiety or fear. Instead, many described a broader emotional landscape marked by grief, despair, emotional numbness, and a deep sense of pessimism about existence. These reactions were rarely experienced as sudden or separate; rather, participants spoke of them as gradually accumulating over time. Climate-related stressors were often perceived as intensifying and reinforcing existing depressive emotional patterns, rather than introducing entirely new feelings. In this sense, the climate crisis was experienced as an emotional amplifier—deepening sadness, hopelessness, and psychological distress within the ongoing experience of living with depression.

### Theme 5: coping mechanisms and recommendations

Participants described a wide range of coping strategies that they perceived as helpful in dealing with the emotional and psychological burden of the climate crisis. These strategies reflected both individual efforts to regulate distress and broader expectations that responsibility should be shared at societal and institutional levels. Coping was not described as a single or uniform process; rather, participants’ responses varied and included both adaptive, resilience-oriented strategies and more ambivalent or avoidant approaches.

At the *individual level*, social support emerged as one of the most important and valued coping resources. Many participants emphasized that spending time with family members or friends helped them feel less isolated and emotionally overwhelmed, particularly when climate-related concerns intensified their depressive symptoms.

*“When I feel bad*,* I seek help from my friends or family. Social support helps me cope better.” (DP11)*.

Engaging in physical activity was another commonly described way of coping. Participants noted that activities such as exercise or spending time in nature offered both physical relief and a sense of psychological grounding, even if motivation was sometimes low.

*“I try to take care of my health; I do sports.” (DP10)*.

Similarly, one participant described selectively engaging with outdoor activities depending on weather conditions:

*“I usually don’t feel like doing much when the weather is gloomy… but when I go on nature walks*,* I enjoy it.” (DP1)*.

Participants also described cognitive and emotional strategies aimed at managing distress. Spiritual practices, particularly prayer, were mentioned as a source of comfort, hope, and emotional reassurance in the face of uncertainty.

*“I pray that the climate crisis will not affect my future. I pray that we can experience the seasons properly.” (DP6)*.

Some participants acknowledged using avoidance or deliberate disengagement as a short-term way of coping, while at the same time recognizing that this strategy did not resolve their concerns.

“*I usually try not to think about it*,* but I know this is not a solution.” (DP4)*.

Others described coping as an ongoing internal struggle, emphasizing that seeking help became especially important when their own coping resources felt insufficient.

*“I fight on my own as much as I can. When I feel bad*,* I ask for help. When I find help*,* I feel better.” (DP11)*.

Medical and professional support also played a role in participants’ coping efforts. A few described adherences to pharmacological treatment as part of their strategy for managing the emotional impact of climate-related stressors.

*“I go to the doctor and receive medication… witnessing these really bad things affects me deeply. The medication helps a bit.” (DP2)*.

Together, these accounts suggest that participants actively sought ways to manage climate-related distress by drawing on social, behavioral, spiritual, and medical resources. While participants varied in how effective they perceived these strategies to be, many described them as helping to preserve emotional stability and a sense of agency in the face of ongoing challenges.

Beyond individual coping, participants also emphasized the importance of collective and institutional responsibility in addressing the climate crisis. Some described engaging in environmentally responsible behaviors as a way of regaining a sense of control and contributing, even in small ways, to mitigation efforts.

*“I try to act in more environmentally sensitive ways—like not littering*,* using public transportation when possible*,* saving water*,* and managing waste properly.” (DP5)*.

At the same time, many participants expressed frustration that individual efforts felt insufficient without broader systemic change. They articulated clear expectations toward governments, institutions, and policymakers, emphasizing the need for concrete action and regulation.

*“We expect our government to take action. Pollution needs to be prevented.” (DP9)*.

Another participant similarly highlighted the role of industrial pollution:

*“Our world is being damaged by exhaust fumes and factory emissions… these need to be prevented.” (DP10)*.

Some participants also suggested that increasing public awareness could play a meaningful role in addressing the climate crisis, noting that many people may not fully understand its consequences.

*“Maybe I could participate in activities that create awareness. I think many people are not sufficiently informed about these issues.” (DP5)*.

Taken together, participants’ accounts suggest that coping with the climate crisis was experienced not only as an individual psychological task but also as a shared social and institutional concern. Although depressive symptoms sometimes made coping more difficult, many participants nonetheless described efforts to endure, adapt, and regain a sense of agency. Through maintaining social connections, engaging in meaning-making practices, caring for their health, and advocating for environmental responsibility, participants demonstrated not only vulnerability but also ongoing attempts to live with and respond to climate-related distress while managing the challenges of depression.

Across the themes, participants’ accounts reveal that the climate crisis was experienced as a pervasive and deeply interconnected stressor that shaped many aspects of everyday life. Rather than being seen as a series of isolated events, climate-related changes were described as accumulating over time and interacting with participants’ existing depressive vulnerabilities. Disruptions in daily routines and social functioning were closely intertwined with health concerns, emotional reactions, and worries about the future. For many participants, physical discomfort—such as extreme heat or sleep disturbance—and visible environmental degradation gave rise to emotional distress, which in turn increased social withdrawal and made coping feel more difficult. Climate-related anxiety and future-oriented concerns often intensified feelings of hopelessness and ecological grief, while emotional responses such as restlessness, sadness, and emotional numbness further limited daily functioning. At the same time, participants spoke about various ways of coping with these challenges, including seeking social support, engaging in health-related or spiritual practices, and adopting environmentally responsible behaviors. Although these strategies did not remove distress entirely, they were experienced as helping participants preserve a sense of agency, connection, and meaning in the face of ongoing uncertainty. Taken together, the findings suggest that participants experienced the climate crisis as an ongoing, embodied, and relational part of their lives, in which daily routines, health, emotions, future expectations, and coping efforts were closely intertwined rather than separate or sequential experiences.

## Discussion

This study explored the lived experiences of individuals diagnosed with Major Depressive Disorder (MDD) regarding the perceived psychological, emotional, and daily-life impacts of the climate crisis. Using a phenomenological approach, the findings highlight how participants perceived climate-related changes as intersecting with their depressive symptoms, daily functioning, health concerns, emotional responses, and coping strategies. Rather than implying a direct causal relationship, the results reflect participants’ subjective interpretations and lived experiences of climate-related stressors.

The findings of this study align with Lazarus and Folkman’s stress–appraisal theory and diathesis–stress models of depression, suggesting that climate-related stressors are experienced through ongoing appraisal processes shaped by underlying vulnerability. From this perspective, individuals’ emotional and behavioral responses to stressors are shaped by how environmental demands are cognitively appraised and by the perceived availability of coping resources^22^. Participants in the present study commonly described climate-related changes—such as extreme heat, environmental degradation, and uncertainty about the future—as overwhelming and beyond their control, experiences that were closely associated with heightened feelings of helplessness, hopelessness, and psychological distress. In line with current findings, recent systematic and integrative reviews have shown that climate-related stressors and eco-anxiety are strongly associated with depressive symptoms, feelings of powerlessness, and reduced psychological well-being, espescially when individuals perceive lack of coping mechanism to respond effectively to environmental threats^23,24^.

Viewed through a diathesis–stress approach^25^, pre-existing vulnerabilities related to depression, including negative cognitive biases, rumination, and reduced psychological resilience, may increase sensitivity to climate-related stressors. Participants’ narratives suggest that climate-related threats were not experienced as initiating depressive symptoms, but rather as factors that intensified and sustained existing symptom patterns. This interpretation is consistent with contemporary models that emphasize bidirectional and interactive processes between environmental stressors and mental health, rather than simple unidirectional causality. Early conceptual work has similarly highlighted that climate change represents not only an environmental challenge but also a significant psychosocial stressor with implications for mental health^26,27^. The present findings build on this literature by offering qualitative insight into how individuals with a clinical diagnosis of MDD perceive and make sense of climate-related stressors within the context of their illness.

In the current study, participants described disruptions in daily routines, reduced motivation, and social withdrawal in response to climate-related stressors. These experiences are consistent with broader literature showing that climate-related distress is associated with functional impairments and reduced quality of life in general populations^28^, as well as earlier studies suggesting that climate change can undermine social cohesion and daily functioning^27,29^.

Importantly, participants did not describe climate change as a direct cause of depression; rather, they perceived climate-related uncertainty and pessimism as amplifying existing difficulties in maintaining social roles, routines, and interpersonal engagement. In a recent systematic analysis, eco-anxiety has been found to have positive correlations with mental health outcomes, including psychological distress, depressive symptoms, anxiety symptoms, and stress^23^.

Participants reported perceived negative effects of the climate crisis on both physical and mental health, including fatigue, sleep disturbances, and worsening mood symptoms. These accounts align with empirical evidence linking rising temperatures and environmental stressors to increased mental health emergencies, mood disturbances, and sleep problems^30,31^. Individuals with MDD may be particularly sensitive to such stressors due to characteristic affective and cognitive vulnerabilities. Individuals with MDD may be particularly sensitive to such stressors due to characteristic affective and cognitive vulnerabilities, including reduced perceived control, which may amplify emotional responses to climate-related threats.

Consistent with existing research, participants described fluctuations in depressive symptoms during extreme weather conditions, suggesting that climate-related exposures may act as contextual stressors that intensify symptom burden rather than independent etiological factors. Previous research has also suggested that climate-related stressors may complicate treatment processes and negatively influence quality of life among individuals with mental disorders^26,32,33^. The present findings add qualitative depth to this literature by showing how individuals with MDD interpret these challenges as barriers to recovery rather than as independent causes of their illness.

A central theme involved climate anxiety and future-related concerns as participants expressed fear, uncertainty, ecological grief, and a sense of powerlessness regarding environmental degradation and future generations. These emotional responses are consistent with growing literature on eco-anxiety and climate-related distress, which has been associated with depressive symptoms, psychological distress, and reduced well-being^24^.

While eco-anxiety has been extensively studied in general and youth populations, this study contributes by highlighting how individuals with MDD perceive climate-related anxiety through the lens of their illness, often describing intensified hopelessness and pessimism consistent with depressive cognitive schemas. Feelings of sadness, loss, and grief related to environmental degradation were prominent in participants’ narratives and resonate with the concept of ecological grief, which describes mourning reactions to environmental loss and biodiversity degradation^34^. For individuals with MDD, such emotional responses may be intensified by depressive cognitive patterns, including hopelessness and rumination, shaping how climate-related losses are experienced and processed.

Participants reported a range of coping strategies, including seeking social support, engaging in physical activity, spiritual practices, medical treatment adherence, and pro-environmental behaviors. These strategies reflect coping patterns identified in previous climate-related research^35^and align with Lazarus’s conceptualization of coping as cognitive, emotional, and behavioral efforts to manage stressors.

Notably, participants’ coping strategies were shaped by cultural, social, and personal resources, underscoring the importance of contextually sensitive interventions. While some coping responses appeared adaptive, others (e.g., denial or withdrawal) may reflect maladaptive responses associated with depressive symptoms.

The findings of this study should be read within the specific climatic, socioeconomic, and cultural context of Eastern Turkey, where the research was conducted. Van and its surrounding region are characterized by harsh continental climate conditions, including cold winters, hot summers, increasing temperature variability, and growing concerns related to drought and water scarcity. These environmental conditions directly affect agriculture, animal husbandry, and daily living conditions, which constitute key livelihood sources in the region. As a result, climate-related changes may be experienced not as abstract global threats but as immediate and tangible stressors influencing daily life. Socioeconomic factors may have further shaped participants’ perceptions of the climate crisis. Compared to more economically developed regions, Eastern Turkey faces higher levels of economic vulnerability, limited access to resources, and fewer adaptive infrastructures. Participants’ concerns about food security, access to basic resources, and future uncertainty should therefore be understood in relation to these structural conditions. For individuals living with depression, such contextual vulnerabilities may intensify feelings of helplessness and pessimism, particularly when environmental changes are perceived as uncontrollable and unavoidable. Cultural context also played a role in shaping participants’ experiences and coping responses. Strong family ties, community-oriented values, and spiritual or religious practices are prominent in the region and were reflected in participants’ reliance on social support and spirituality as coping strategies. These culturally embedded resources appeared to offer emotional comfort and meaning-making in the face of climate-related distress, even when practical solutions felt limited.

### Strengths and limitations

To our knowledge, no prior qualitative study has focused exclusively on individuals with a clinical diagnosis of MDD to explore their lived experiences of the climate crisis. While previous research has linked climate change to depression and psychological distress, most studies have relied on quantitative designs or non-clinical samples. By using in-depth individual interviews, this study provides rich, first-person insights into how people living with depression perceive, experience, and make sense of climate-related stressors in daily life.

Several limitations should be acknowledged. Due to the phenomenological and cross-sectional design, the findings reflect subjective experiences and do not allow for causal relationship. The results suggest a bidirectional relationship in which climate-related stressors may exacerbate depressive symptoms, while depressive cognitive and emotional patterns may also influence perceptions of climate-related threats. The sample consisted of a small group of relatively stable outpatients recruited from a single clinical center, which may limit transferability to individuals with more severe symptoms or different clinical and cultural contexts.

In addition, the cross-sectional design precludes examination of changes over time, and reliance on self-reported experiences introduces the possibility of recall bias or mood-related influences. Although strategies such as reflexive analysis, member checking, and external review were used to enhance analytic rigor, researcher influence cannot be fully eliminated. Despite these limitations, the findings may be informative for similar contexts characterized by climate vulnerability and limited adaptive resources. Future research using longitudinal and multi-center designs is warranted to further explore the mental health impacts of climate change among individuals with depression.

## Conclusion

This study provides qualitative evidence on how individuals with a clinical diagnosis of Major Depressive Disorder perceive and experience the climate crisis in their daily lives. By focusing on lived experiences rather than symptom measures, the findings highlight how climate-related stressors are understood as emotionally, physically, and socially meaningful within the context of depression. The study extends existing climate–mental health research by foregrounding a clinically vulnerable population and offers insights that may inform mental health practice and policy approaches sensitive to environmental and contextual stressors.

From the participants’ perspectives, the climate crisis was experienced as a contextual stressor shaping multiple aspects of daily life. Climate-related conditions were perceived as aggravating depressive symptoms, disrupting routines, and contributing to physical discomfort, emotional strain, and heightened anxiety—particularly regarding uncertainty about the future. Alongside these challenges, participants described various coping strategies, including seeking social support, engaging in physical activity, drawing on spirituality, and adhering to treatment, while also emphasizing the importance of institutional responsibility and public awareness.

By foregrounding the lived experiences of individuals with a clinical diagnosis of depression, this study offers in-depth qualitative insight into how climate-related stressors are interpreted and integrated into personal illness narratives. The findings suggest a perceived need for supportive resources, preventive strategies, and mental health services that are sensitive to environmental and contextual stressors. Taken together, the results highlight the value of incorporating patients’ climate-related concerns into clinical practice, policy discussions, and future research, without implying direct causal relationships.

## Data Availability

The datasets generated and/or analysed during the current study are not publicly available due [it includes personal experiences] but are available from the corresponding author on reasonable request.

## References

[1] Calvin, K. et al. *IPCC, 2023: Climate Change 2023: Synthesis Report. Contribution of Working Groups I, II and III to the Sixth Assessment Report of the Intergovernmental Panel on Climate Change [Core Writing Team, H. Lee and J. Romero (eds.)]. IPCC, Geneva, Switzerland.* (P. Arias, M. Bustamante, I. Elgizouli, G. Flato, M. Howden, C. Méndez-Vallejo, J. J. Pereira, R. Pichs-Madruga, S. K. Rose, Y. Saheb, R. Sánchez Rodríguez, D. Ürge-Vorsatz, C. Xiao, N. Yassaa, J. Romero, J. Kim, E. F. Haites, Y. Jung, R. Stavins, … C. Péan, Eds.). (2023). 10.59327/IPCC/AR6-9789291691647

[2] Rocha, J., Oliveira, S., Viana, C. M. & Ribeiro, A. I. Climate change and its impacts on health, environment and economy. In One Health (253–279). (Elsevier, 2022).

[3] Charlson, F. et al. Climate change and mental health: a scoping review. *Int. J. Environ. Res. Public Health*. **18** (9), 4486 (2021). 33922573 10.3390/ijerph18094486 PMC8122895

[4] Clayton, S. Climate anxiety: psychological responses to climate change. *J. Anxiety Disord.* **74**, 102263 (2020). 32623280 10.1016/j.janxdis.2020.102263

[5] Crane, K., Li, L., Subramanian, P., Rovit, E. & Liu, J. Climate change and mental health: A review of empirical evidence, mechanisms and implications. *Atmosphere*, *13*(12), 2096. (2022a). 10.3390/atmos13122096 PMC10508914 37727770

[6] Leal Filho, W. et al. Climate change, extreme events and mental health in the Pacific region. *Int. J. Clim. Change Strateg. Manag.* **15** (1), 20–40 (2023).

[7] Nori-Sarma, A. et al. Association between ambient heat and risk of emergency department visits for mental health among US adults, 2010 to 2019. *JAMA Psychiatry*. **79** (4), 341–349 (2022). 35195664 10.1001/jamapsychiatry.2021.4369 PMC8867392

[8] Dobkins, K. R., Dickenson, J., Lindsay, D. & Bondi, T. *Mental Health and Resilience in the Climate Crisis: A case study of well-being courses targeting college students*. (2023). 10.3389/fpubh.2023.1175594 PMC10413109 37575115

[9] Hickman, C. et al. Climate anxiety in children and young people and their beliefs about government responses to climate change: a global survey. *Lancet Planet. Health*. **5** (12), e863–e873 (2021). 34895496 10.1016/S2542-5196(21)00278-3

[10] White, B. P. et al. Mental health impacts of climate change among vulnerable populations globally: an integrative review. *Annals Global Health*, **89**(1). (2023). 10.5334/aogh.4105 PMC10558031 37810609

[11] Ogunbode, C. A. et al. Negative emotions about climate change are related to insomnia symptoms and mental health: Cross-sectional evidence from 25 countries. *Current Psychol.*, 1–10. (2021).

[12] Wigand, M. E., Timmermann, C., Scherp, A., Becker, T. & Steger, F. Climate change, pollution, deforestation, and mental health: Research trends, gaps, and ethical considerations. *GeoHealth*,** 6** (11), e2022GH000632. (2022). 10.1029/2022GH000632 PMC9623432 36330078

[13] Corvalan, C. et al. Mental health and the global climate crisis. *Epidemiol. Psychiatric Sci.* **31**, e86 (2022). 10.1017/S2045796022000361 PMC9762138 36459133

[14] de Jarnette, J. Climate change psychological distress: an underdiagnosed cause of mental health disturbances. *J. Am. Board. Family Med.* **37** (1), 11–14 (2024). 10.3122/jabfm.2023.230169R1 38448240

[15] Yıldırım, A. et al. Türkiye’den İklim Krizine Betimsel Metotlar Ile Kısa Bir Bakış. *Ankara Üniversitesi Sosyal Bilimler Dergisi*. **3** (1), 1–10. 10.1080/2159676X.2019.1628806 (2023).

[16] Tong, A., Sainsbury, P. & Craig, J. Consolidated criteria for reporting qualitative research (COREQ): a 32-item checklist for interviews and focus groups. *Int. J. Qual. Health Care*. **19** (6), 349–357 (2007). 17872937 10.1093/intqhc/mzm042

[17] Braun, V. & Clarke, V. Reflecting on reflexive thematic analysis. *Qualitative Res. Sport Exerc. Health*. **11** (4), 589–597. 10.1080/2159676X.2019.1628806 (2019).

[18] Creswell, J. W., Hanson, W. E., Plano, C., Morales, A. & V. L., & Qualitative research designs: selection and implementation. *Couns. Psychol.* **35** (2), 236–264 (2007).

[19] Hennink, M. & Kaiser, B. N. Sample sizes for saturation in qualitative research: A systematic review of empirical tests. *Soc. Sci. Med.* **292**, 114523 (2022). 34785096 10.1016/j.socscimed.2021.114523

[20] Ahmad, S. & Senese, V. P. Exploring patient-centered interventions and comorbid symptoms in episodic migraine management: A qualitative study. *Curr. Psychol.* **44** (19), 15538–15547. 10.1007/s12144-025-08283-z (2025).

[21] Lincoln, Y. & Guba, B. *Naturalistic Inquiry. Beverly Hills: SAGE Publikations* (Inc, 1985).

[22] Lazarus, R. & Folkman, S. Stress and coping. *New. York*. **18** (31), 34–42 (1985).11658563

[23] Cosh, S. M. et al. The relationship between climate change and mental health: a systematic review of the association between eco-anxiety, psychological distress, and symptoms of major affective disorders. *BMC Psychiatry*. **24** (1), 833 (2024). 39567913 10.1186/s12888-024-06274-1 PMC11577747

[24] Gianfredi, V. et al. Climate change perception and mental health. Results from a systematic review of the literature. *Eur. J. Invest. Health Psychol. Educ.* **14** (1), 215–229 (2024).10.3390/ejihpe14010014. 10.3390/ejihpe14010014 PMC10814599 38248134

[25] Monroe, S. M. & Simons, A. D. Diathesis-stress theories in the context of life stress research: implications for the depressive disorders. *Psychol. Bull.* **110** (3), 406 (1991). 1758917 10.1037/0033-2909.110.3.406

[26] Berry, H. L., Bowen, K. & Kjellstrom, T. Climate change and mental health: a causal pathways framework. *Int. J. Public. Health*. **55**, 123–132 (2010). 20033251 10.1007/s00038-009-0112-0

[27] Fritze, J. G., Blashki, G. A., Burke, S. & Wiseman, J. Hope, despair and transformation: climate change and the promotion of mental health and wellbeing. *Int. J. Mental Health Syst.* **2**, 1–10 (2008). 10.1186/1752-4458-2-13 PMC2556310 18799005

[28] Heeren, A., Mouguiama-Daouda, C. & Contreras, A. On climate anxiety and the threat it May pose to daily life functioning and adaptation: A study among European and African French-speaking participants. *Clim. Change*. **173** (1), 15 (2022). 35912274 10.1007/s10584-022-03402-2 PMC9326410

[29] Miguel, E., Satyanath, S. & Sergenti, E. Economic shocks and civil conflict: an instrumental variables approach. *J. Polit. Econ.* **112** (4), 725–753 (2004).

[30] Corvetto, J. F. et al. Impact of heat on mental health emergency visits: A time series study from all public emergency centres, in Curitiba, Brazil. *BMJ Open.* **13** (12). 10.1136/bmjopen-2023-079049 (2023). 10.1136/bmjopen-2023-079049 PMC10748883 38135317

[31] Crane, K., Li, L., Subramanian, P., Rovit, E. & Liu, J. Climate change and mental health: A review of empirical evidence, Mechanisms and Implications. *Atmosphere* **13**, 12. (MDPI. 2022b). 10.3390/atmos13122096 10.3390/atmos13122096 PMC10508914 37727770

[32] Cianconi, P., Betrò, S. & Janiri, L. The impact of climate change on mental health: a systematic descriptive review. *Front. Psychiatry*. **11**, 490206 (2020). 10.3389/fpsyt.2020.00074 PMC7068211 32210846

[33] Clayton, S., Manning, C., Krygsman, K. & Speiser, M. *Mental Health and our Changing Climate: Impacts, implications, and Guidance* (American Psychological Association and EcoAmerica, 2017).

[34] Cunsolo, A. & Ellis, N. R. Ecological grief as a mental health response to climate change-related loss. *Nat. Clim. Change*. **8** (4), 275–281 (2018).

[35] Mah, A. Y. J., Chapman, D. A., Markowitz, E. M. & Lickel, B. Coping with climate change: three insights for research, intervention, and communication to promote adaptive coping to climate change. *J. Anxiety Disord.* **75**, 102282 (2020). 32781413 10.1016/j.janxdis.2020.102282

